# Exploration of Baicalein-Core Derivatives as Potent Antifungal Agents: SAR and Mechanism Insights

**DOI:** 10.3390/molecules28176340

**Published:** 2023-08-30

**Authors:** Heyang Zhou, Niao Yang, Wei Li, Xuemi Peng, Jiaxiao Dong, Yuanying Jiang, Lan Yan, Dazhi Zhang, Yongsheng Jin

**Affiliations:** 1 School of Pharmacy, Naval Medical University, Shanghai 200433, China; zhy19930701@outlook.com (H.Z.); ylansmmu@sina.com (L.Y.); 2 School of Pharmacy, Anhui Medical University, Hefei 230022, China; 3 Department of Pharmacy, Shanghai Tenth People’s Hospital, School of Medicine, Tongji University, Shanghai 200072, China; jiangyy@tongji.edu.cn

**Keywords:** baicalein, **BE**-core, antifungal, biofilm, *o*-dihydroxyls, *vic*-trihydroxyls

## Abstract

**Highlights:**

**What are the main findings?**

- Baicalein-Core Derivatives were designed and synthesized as Potent Anti-Fluconazole-resistant fungal Agents.
- *O*-dihydroxyls and *vic*-trihydroxy groups on either the A ring or B ring of flavones play a crucial role.
- MoA: Inhibit hypha formation; Little effect on ergosterol biosynthesis; Weak inhibitory effect on Eno1.
- Potential targets: 1,3-β-d-glucan synthase catalytic subunit, 1,3-β-d-glucan-UDP glucosyltransferase, and glycosyl-phosphatidylinositol protein by in-silico assay.

**What is the implication of the main finding?**

- Provide potential synergistic antifungals with new MoA.

**Abstract:**

Baicalein (**BE**), the major component of *Scutellaria Baicalensis*, exhibited potently antifungal activity against drug-resistant *Candida albicans*, and strong inhibition on biofilm formation. Therefore, a series of baicalein-core derivatives were designed and synthesized to find more potent compounds and investigate structure–activity relationship (SAR) and mode of action (MoA). Results demonstrate that **A4** and **B5** exert a more potent antifungal effect (MIC_80_ = 0.125 μg/mL) than **BE** (MIC_80_ = 4 μg/mL) when used in combination with fluconazole (FLC), while the MIC_80_ of FLC dropped from 128 μg/mL to 1 μg/mL. SAR analysis indicates that the presence of 5-OH is crucial for synergistic antifungal activities, while *o*-dihydroxyls and *vic*-trihydroxyls are an essential pharmacophore, whether they are located on the A ring or the B ring of flavonoids. The MoA demonstrated that these compounds exhibited potent antifungal effects by inhibiting hypha formation of *C. albicans*. However, sterol composition assay and enzymatic assay conducted in vitro indicated minimal impact of these compounds on sterol biosynthesis and Eno1. These findings were further confirmed by the results of the in-silico assay, which assessed the stability of the complexes. Moreover, the inhibition of hypha of this kind of compound could be attributed to their effect on the catalytic subunit of 1,3-β-d-glucan synthase, 1,3-β-d-glucan-UDP glucosyltransferase and glycosyl-phosphatidylinositol protein, rather than inhibiting ergosterol biosynthesis and Eno1 activity by Induced-Fit Docking and Molecular Dynamics Simulations. This study presents potential antifungal agents with synergistic effects that can effectively inhibit hypha formation. It also provides new insights into the MoA.

## 1. Introduction

Invasive fungal infections (IFIs) result in high levels of sickness and death among individuals with weakened immune systems, such as those with AIDS, receiving cancer chemotherapy, or undergoing immunosuppressive therapy [1]. *C. albicans* is a common pathogen that causes life-threatening infections in these patients [2]. The frontline treatment for systemic *C. albicans* infections is Fluconazole (FLC). Unfortunately, over the past few decades, traditional antifungal drugs have become less effective against drug-resistant fungi, creating a dire situation for humans [3,4]. Consequently, the development of novel antifungal drugs is urgently needed. One promising approach to overcome azole resistance involves sensitizing *C. albicans* using small molecules, such as arthro-colins [5], 1,3,5-triazines derivatives [6], and natural products [7,8,9,10,11].

*C. albicans* can form adherent biofilms on various surfaces in vitro, leading to device-related infections [12]. The presence of hypha plays a crucial role in the biofilm formation of *C. albicans*. This unique structural feature allows the fungus to withstand the host’s immune defense and enhances its resistance to antifungal drugs [13]. Consequently, targeting biofilm is an ideal strategy for antifungal drugs. Echinocandin is a successful example, as it exhibits better selectivity and lower toxicity by specifically targeting fungal biofilms [14].

Flavonoids are a group of natural polyphenol substances widely distributed in plants, such as vegetables and fruits. in daily diet. A variety of biological activities have been reported, including anti-inflammatory, anti-tumor, hypolipidemic, anti-fungal and viral [15]. Baicalein (**BE**, 5,6,7-trihydroxyflavone) is the major constituent in plants of *Scutellaria baicalensis*. The roots of *S. baicalensis,* known as Huang qin, have been widely used in traditional Chinese medicines for thousands of years to treat common colds, fever and other respiratory disorders.

Our previous study found that **BE** had strong antifungal activity against drug-resistance fungus when used alone or in combination with FLC. The MIC_80_ of FLC decreases from >64 μg/mL when used alone to ≤0.125–2 μg/mL when used in combination with 2–16 μg/mL of **BE**. Additionally, it could be observed that the biofilm of *C. albicans* was inhibited in a dose dependent manner at concentrations between 4–32 μg/mL [16,17]. Studies on mode of action (MoA) of **BE** revealed that **BE** could exert antifungal activity through inhibition of hypha formation and glycolysis disruption, which demonstrated that **BE** could be multi-target [18].

**Figure 1:**
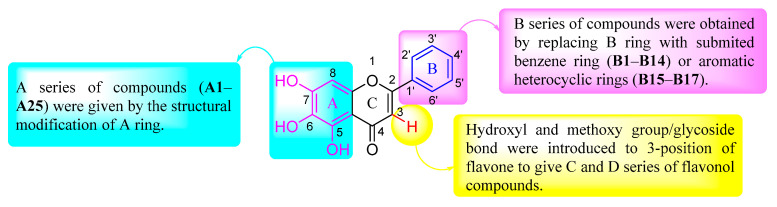
General structures of **BE** analogues.

Therefore, **BE** is an ideal lead compound used as a sensitizer for drug-resistant fungus in combination with FLC. However, as far as we know, there is a lack of systemic study on **BE** or flavonoids regarding their antifungal activity and structure–activity relationship. Inspired by the unique *vic*-trihydroxy-4*H*-chromen-4-one structure of **BE**, a series of natural flavonoids were screened for their alone and synergistic antifungal activity with FLC, and based on these results a series of **BE** derivatives were further designed, synthesized and screened for their antifungal activities, alone and in combination with FLC. Their possible MoA was also investigated. The SAR study based on the structure of **BE** was carried out according to the strategy shown in Figure 1. Based on these findings, our objective is to identify potential compounds that possess strong synergistic antifungal effects, particularly in inhibiting hypha formation. Furthermore, we aim to investigate the possible mechanisms of action (MoA) underlying these effects.

## 2. Results

### 2.1. Molecular Design of **BE**-Core Derivatives

This study examined the initial structural modification carried on the A ring of **BE**, aiming to evaluate the impact of its distinctive *vic*-trihydroxyls on its antifungal activities. The OH, OMe, NO_2_, NH_2_ or Br groups, etc., were introduced to the A ring to afford corresponding A series of compounds. SAR analysis revealed that the presence of 5-OH is crucial for synergistic antifungal effects, while *o*-dihydroxyls and *vic*-trihydroxyls represent essential pharmacophores. 

Then, the B ring of **BE** was modified by substitution with a hydroxyl or methoxyl group, as well as aromatic heterocyclic rings, to give a B series of compounds. 

**Scheme 1:**
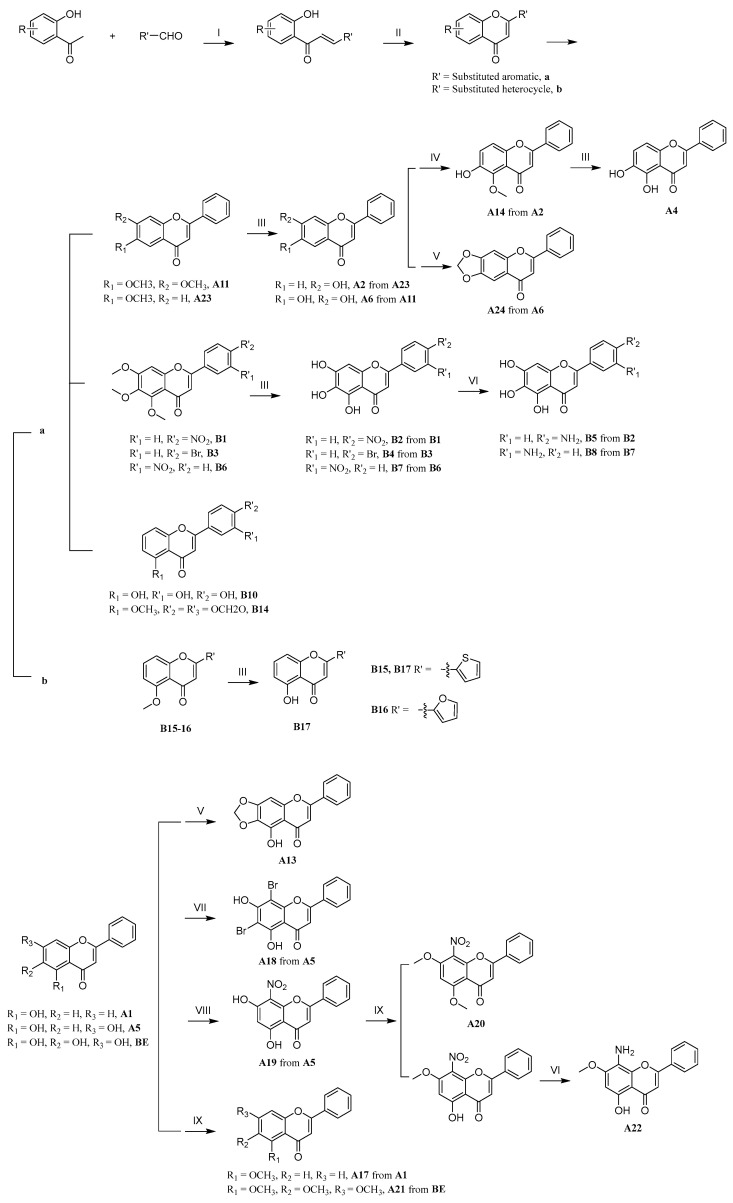
Synthesis of flavones. (I) NaOH, MeOH. (II) I_2_, DMSO. (III) BBr3, DCM. (IV) IBD, CH_3_OH. (V) BrCH_2_Cl. (VI) H_2_, Pd/C. (VII) Br_2_/AcOH. (VIII) HNO_3_. (IX) CH_3_I, K_2_CO_3_, THF.

Additionally, the hydroxyl group was introduced to the 3-position of **BE** to give a C series of flavonol compounds. This alteration aimed to investigate the influence of 3-position substitution on biological activity. The general structures of **BE** analogues are outlined in Figure 1 and the synthesis routes are shown in Scheme 1.

### 2.2. Chemistry

Among these synthetic compounds, **A11, A23**, **B1, B3, B6, B10** and **B14**–**B16** were synthesized by aldol condensation of a mixture of substituted acetophenone with corresponding benzaldehyde in the presence of 20–40% aqueous sodium hydroxide in methanol at room temperature, and the resulting mixture subsequently reacted with iodide in DMSO at reflux at 120–140 °C to yield methoxyl substituted flavone, then demethylation with BBr_3_ gave corresponding hydroxyl substituted flavones. Compounds **A17** and **A20**–**21** were obtained by corresponding flavone reaction with CH_3_I. Compound **A13** was synthesized by reaction of **BE** and BrCH_2_Cl [19]. The 6-hydroxyflavone was oxidized by iodo-benzene diacetate (IBD) according to the reference method, in order to give **A14** [20]. Bromo substituents were introduced to chrysin (**A5**) in the presence of Br_2_ in AcOH to give **A18**. Nitration of chrysin or 5-hydroxyflavone with 70% HNO_3_ in acetic acid gave **A19** and 5-hydroxy-7-methoxy-8-nitroflavone [21], then 5-hydroxy-7-methoxy-8-nitroflavone was reduced by catalytic hydrogenation to give **A22**.

A total of 46 important intermediates and 42 compounds were synthesized with yield ranging from 62% to 78%. All other tested compounds were isolated and identified from plants in our lab or commercially available. The structures of synthetic compounds were confirmed by spectroscopic data (NMR and MS) and satisfactory purity was obtained for all the compounds. In total, 62 compounds were screened for their antifungal activity.

### 2.3. In Vitro Synergistic Antifungal Activities and SAR

The in vitro synergistic antifungal activities of **BE** analogues were tested using the micro broth dilution method, according to the standards of the National Committee Clinical and Laboratory Standards Institute, USA [22]. When FLC-resistant *C. albicans* was treated with FLC or **BE** analogues, the individual MIC_80_ (the lowest concentration of the agents that inhibited growth by 80%) values were determined. Furthermore, the fractional inhibitory concentration index (FICI) of each agent was calculated by determining the ratio of the interaction of MIC_80_/individual MIC_80_. The interaction modes, either synergistic or indifferent, were defined according to FICI values of ≤0.5 or ≥1, respectively [23]. The MIC_80_ of FLC against the FLC-resistant *C. albicans* (clinical isolate 103) was determined to be 128.0 μg/mL. In this assay, the final concentration of FLC (FICI, in Table 1, Table 2, Table 3 and Table 4) in each well was fixed at a single value (8.0 μg/mL), whereas **BE** analogues were diluted into a series of gradient concentrations (0.25–64.0 μg/mL). 

As shown in Table 1, **BE** showed potent synergistic activity in combination with FLC (MIC_80_ = 8.0 μg/mL, FICI = 0.313). Flavonoids of series A were designed to investigate the effects of the type, number, and position of the substituents on the A ring on the activity. The results demonstrated that, except for **A5** and **A22**, all other flavones with 5-OH (**A1**, **A4**, **A8**–**A10**, **A12**–**13**, **A16**, **A18**–**19**, and **A25**) showed synergistic antifungal activity when used in combination with 8 μg/mL of FLC. Among them, the MIC values of **A1**, **A4**, **A8**, **A9**, **A12**–**13**, **A16** and **A18** were all less than or equal to that of **BE**. Compounds **A5** (5,7-dihydroxyflavone) and **A22** (8-amino-5-hydroxy-7-methoxyflavone) did not show antifungal activity, which could be due to meta-disubstituted (5,7-dihydroxy), whereas, if 5-OH was changed into 5-OMe or H, all the compounds (**A2**, **A3**, **A14**, **A15**, **A17**, **A20**, **A21** and **A23**) showed weak or no antifungal activity. Interestingly, compounds **A6**, **A7** and **A24**, from which 5-OH was absent, also exerted comparable antifungal activity on **BE**. An obvious feature of the structures of **A6** and **A7** is that they all possess the *o*-dihydroxy group. **A8**, another *O*-dihydroxy flavone (5,6-dihydroxy-7-methoxyflaonve), which was obtained by methylation of 7-OH of **BE**, also showed equivalent activity to **BE**. Thus, 5-OH or *o*-dihydroxyl moiety on the A ring of flavone is favored for synergistic antifungal activity. Our previous studies also demonstrated that 5-OH or *o*-dihydroxy moiety were important pharmacophore [9,24]. Furthermore, bromo, methoxy, nitro or amino group were introduced to position 6 and 8 of **BE** to give **A18**–**A20** to investigate their effects on the antifungal activity. **A18** and **A19,** with bromo or nitro group linked to position 6 or 8, showed similar activity to **BE**. **A20,** with only 5,7-dimethoxyl, and 8-nitro group and **A21** with only methoxyls showed poor or no activity. Thus, introducing of bromo, nitro, amino or methoxy groups to the A ring of flavone could not increase the synergic antifungal activity.

Based on modification on ring A, the effect of substituents on ring B on antifungal activity was investigated. The results are shown in Table 2. We firstly retained the structure of the A ring of **BE**, then introduced nitro group, bromo, amino group or hydroxyl to 3′ or 4′ position of the B ring to obtain **B2**, **B4**, **B5** and **B7**–**B9**. It was found that, except for **B9** (4′-hydroxybaicalein), all the other five flavones (**B2**, **B4**, **B5**, **B7** and **B8**) showed superior or comparable anti-fungal activity to **BE**. Even compounds **B4** (4′-bromobaicalein), **B5** (4′-aminobaicalein) and **B8** (3′-aminobaicalein) showed stronger antifungal activity alone than **BE** (MIC_80_ = 32, 16 and 16 μg/mL for **B4**, **B5** and **B6**). However, methylation of all the hydroxyl groups of the A-ring resulted in complete loss of activities (**B1**, **B3** and **B6**, MIC > 64 μg/mL). Changing 5,6,7-trihydroxyl into the 5-hydroxyl or 5,7-dihydroxyl of the A ring and introducing 4′-OH (**B13**) or 3′,4′-dihydroxyl (**B10** and **B11**) or 3′-hydroxyl-4′-methoxyl (**B12**) to the B ring led to weak or no activity compared to the A series of flavones and **BE**. This suggested that 5,6,7-trihydroxy moiety on the A ring of **BE** is important for the antifungal activities if introducing hydroxyls into the B ring of flavone. However, if 5-OH is retained and an aromatic heterocyclic ring is used instead of the benzene of the B ring, the compound (**B17**) has strong synergistic activities (MIC_80_ = 8.0 μg/mL), whereas methylation of 5-OH of the B series of compounds caused a complete loss of activities (**B14**–**B16**). These results were consistent with that of the A series of compounds. 

The SAR study of series A and B guided us to design C and D series of flavonols to study the impact of 3-OH or 3-substituted oxy group on the antifungal effect. The results are presented in Table 3. In the A and B series of flavones, compounds with 5,7-dihydroxyl (**A5**, **B11**–**13**) showed weak or no activities. However, in the C series of flavones, compounds with 5,7-dihydroxyl (**C1**–**C4**, **C6**–**C7**) showed moderate or potent activities. This demonstrated that 3-hydroxyl is favored for synergic antifungal effect. One exception, i.e., that **C9** (5,7-dihydroxy-4′-methoxy-8-isopentenylflavonol) has no antifungal activity, could be due to the large hindrance of the 8-position isopentenyl position of **C9**. Additionally, similar results for the importance of 5-hydroxy was also observed in the C series of flavones. Most of the compounds with 5-OMe or 5-H in the C series of flavones were inactive, and the methylated flavones at positions 5 and 7 (**C10**–**C11** and **D1**) have no or only weak activity. When *o*-dihydroxy, or even *vic*-trihydroxy moiety, was introduced to the B-ring to afford **C2** (Quercetin), **C5** (Robinetin), **C7** (Myricetin), **C14** (3′,4′-dihydroxyflavonol) and **C16** (Vincetoxicoside B), regardless of whether there is a 5-hydroxy group in the A-ring, all these compounds demonstrated potent synergic antifungal activity (MIC_80_ value ranging from 2.0 μg/mL to 4 μg/mL). Nevertheless, fisetin (**C12**, 7,3′,4′-trihydroxyflavonol) exhibited weak synergistic antifungal activity (MIC_80_ = 32 μg/mL) as an exception, compared with the structures of **C2**, **C7** and **C16**, **C12,** lacking the 5-OH on the A ring; whereas compared with the structure of **C14**, **C12** has 7-OH on the A ring, while **C14** does not have any substituents on the A ring. Thus, it could be concluded that 7-OH is not necessary, and even not conducive to improving antifungal activity.

In addition, when the 3-OH of flavonol was glycosylated, the corresponding compounds **D2**–**D4** lost their antifungal activity. Furthermore, the only structural difference between **B10** (5,3′,4′-trihydroxyflavone) and **C14** (3′,4′-dihydroxyflavonol), and **B11** (Luteolin, 3′,4′,5,7-tetrahydroxyflavone) and **C2** (5,7,3′,4′-tetrahydroxyflavonol) is the lack of 3-OH in **B10** and **B11**, which resulted in **C2** and **C14** exhibiting more potent antifungal activities (MIC_80_ = 4 μg/mL) than **B10** and **B11** (MIC_80_ = 32 μg/mL). These demonstrated that 3-OH is beneficial for antifungal activity.

To further study the antifungal activity, the most potent compounds **A4**, **B4**, **B5**, **C5** and **C14** were evaluated for their antifungal acidity by a checkerboard microdilution assay. As shown in Table 4, all tested compounds showed potent synergic antifungal activities with MIC_80_ values ranging from 0.125 μg/mL to 8 μg/mL when used in combination with 0.25–1 μg/mL of FLC against FLC-resistant *C. albicans*. **C5** and **C14,** at concentrations of 4–8 μg/mL, were found to decrease the MIC_80_ value of FLC from 128 μg/mL to 0.25 μg/mL. Even at a concentration of 0.125 μg/mL, **A4** and **B5** were able to significantly decrease the MIC_80_ value of FLC from 128 μg/mL to 1 μg/mL. Additionally, **B4** showed more potent antifungal activity when used alone, with a MIC_80_ value of 16 μg/mL, which was four times more potent than **BE** (MIC_80_ = 16 μg/mL, alone). In summary, while **C14** (FICI = 0.0645) and **B4** (FICI = 0.0703) exhibited comparable FICI values to **BE** (FICI = 0.0645), **A4** (FICI = 0.0088), **B5** (FICI = 0.0088) and **C5** (FICI = 0.0332) demonstrated significantly greater synergistic activity than **BE**. To further confirm their synergistic antifungal activity, **A4** and **B5** were selected for testing of their antifungal effects against two additional FLC-resistant clinical isolates: *C. albicans* 938 and 939. The results showed that **A4** and **B5** exhibited significant synergistic antifungal effects against *C. albicans* 938 and *C. albicans* 939 (with a MIC_80_ of 0.25 μg/mL when combined with FLC at 8 μg/mL). These findings indicate that these potent compounds possess broad-spectrum and potent synergistic antifungal activities.

In conclusion, 5-OH or *o*-dihydroxy moiety on the A ring of flavone is favored for synergistic antifungal activity. Methylation of hydroxyl decreased the antifungal activity. When 5-OH is present, introduction of the 3-OH could significantly improve antifungal activity. Overall, the presence of *o*-dihydroxy or *vic*-trihydroxy moiety on either the A or B ring has been found to be favorable to antifungal activity. Introduction of Br or NH_2_ to the B ring of **BE** could enhance antifungal activity when used alone. Thus, it was clear that appropriate structural modification of **BE** could obtain higher active flavonoids with synergistic antifungal activity against FLC-resistant *C. albicans.*

### 2.4. In Vitro Hyphal Formation Assay

**Figure 2:**
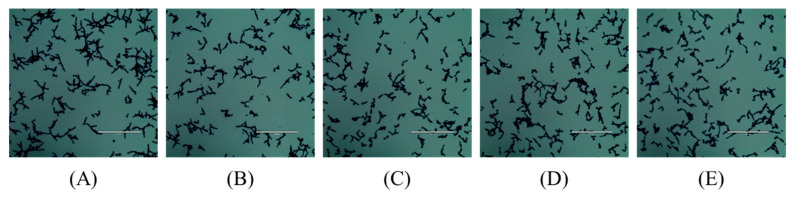
Effects of different concentrations of FLC on the fungi hypha formation. (**A**) Control. (**B**) FLC 2 μg/mL. (**C**) FLC 4 μg/mL. (**D**) FLC 8 μg/mL. (**E**) FLC 16 μg/mL. Scale: 1:100 μm.

**Figure 3:**
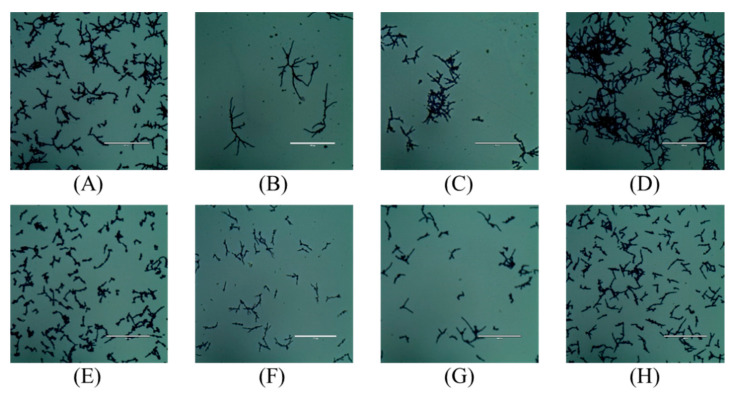
Inhibition of hypha formation. (**A**) Control. (**B**) **A4** 8 μg/mL. (**C**) **B4** 8 μg/mL. (**D**) **B5** 8 μg/mL. (**E**) FLC 16 μg/mL. (**F**) **A4** 8 μg/mL with FLC 8 μg/mL. (**G**) **B4** 8 μg/mL with FLC 8 μg/mL. (**H**) **B5** 8 μg/mL with FLC 8 μg/mL. Scale: 1:100 μm.

In a previous study, **BE** exhibited significant inhibition of fungal biofilm formation [16]. Thereby, compounds **A4**, **B4** and **B5** with the best antifungal activity were selected for in vitro hypha formation assay. The results are presented in Figure 2 and Figure 3. Apparently, FLC did not inhibit the biofilm formation of clinical FLC-resistant *C. albicans* 901 and 904 at the concentrations of 2–16 µg/mL (Figure 2A–E). However, compared to FLC, **A4** and **B4** alone at 8 µg/mL significantly reduced the density of the *C. albicans* (Figure 3B–D). Furthermore, the combination of FLC with compounds **A4**, **B4** and **B5** remarkably inhibited the hypha formation (Figure 3E–H). The density of fungus and the length of hypha were intensely suppressed by compounds **A4**, **B4** and **B5** when used in combination with FLC. This mechanism may contribute to the destruction of the defense of FLC-resistant *C. albicans* and provide an advantageous opportunity for FLC to attack the fungus.

### 2.5. In Vitro Sterol Composition Assay

**Figure 4:**
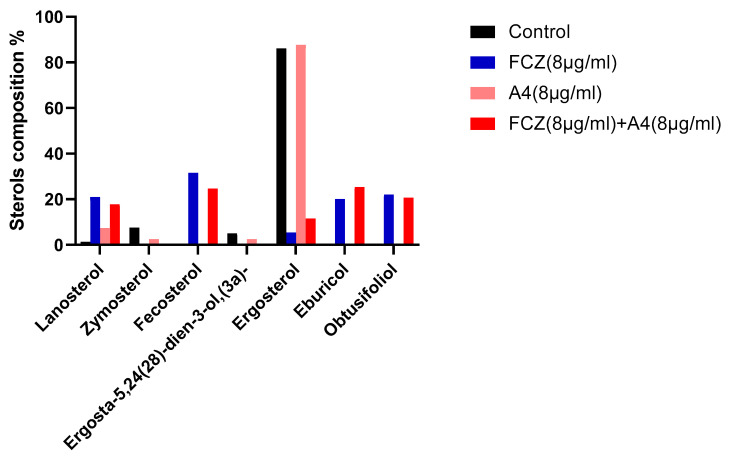
Sterol compositions assay of *C. albicans* after treatment with FLC, compound **A4** and FLC + **A4**.

The potent compound **A4** was evaluated for its impact on the sterol composition in the cell membrane of *C. albicans* through a gas chromatography-mass spectrometry method. Results demonstrated that treatment with FLC at a concentration of 8 μg/mL resulted in a significant reduction of ergosterol in *C. albicans*, while showing elevated levels of eburicol, obtusifoliol and fecosterol. Similarly, in combination with FLC (8 μg/mL) and **A4** (8 μg/mL), the ergosterol decreased, and the eburicol and obtusifoliol increased (Figure 4). However, compound **A4** (8 μg/mL) alone for *C. albicans* did not significantly alter the levels of ergosterol compared to the control group, nor did it increase the levels of eburicol, obstusifoliol, and fecosterol. These findings suggest that compound **A4** did not induce any notable changes in the sterol biosynthesis, indicating that these **BE** derivatives have no impact on the sterol biosynthesis pathway.

### 2.6. In Vitro Eno1 Enzymatic Assay

**Figure 5:**
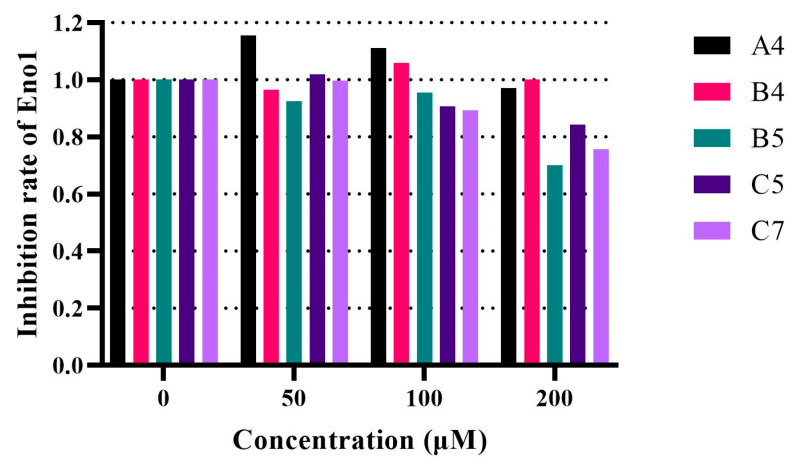
Inhibition of Eno1 enzymatic assay after treatment by **A4**, **B4**, **B5**, **C5**, **C7** at a concentration of 200 μM.

In our previous work, we found that Eno1 is one of the targets of **BE** [18]. Thus, we selected potent compounds **A4**, **B4**, **B5**, **C5** and **C7** for in vitro enzymatic assay. The results are shown in Figure 5. Unexpectedly, these compounds did not exhibit stronger enzyme inhibitory activity than **BE** (IC_50_ = 62.9 ± 2.2 μM). The observed enzyme inhibition activity did not correspond to their in vitro antifungal activity. These results imply the presence of an alternative target for these compounds. Thus, these potent active compounds are worthy of further study on MoA.

### 2.7. In Silico Studies

#### 2.7.1. Molecular Dynamic Simulation

**Figure 6:**
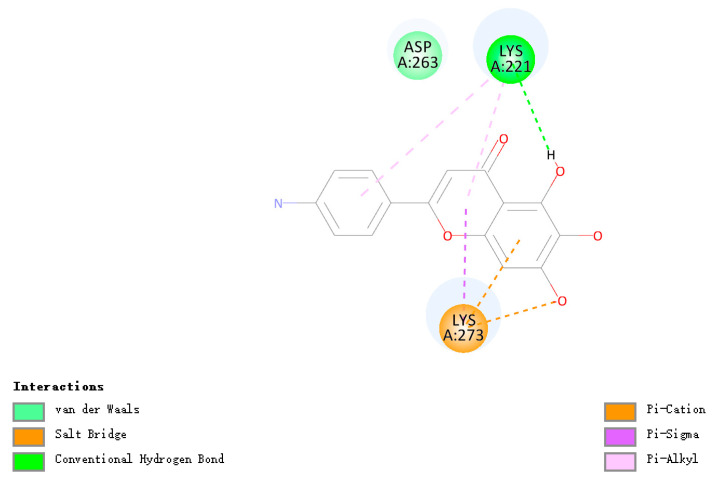
2D diagram of the interaction between **B5** and Eno1.

**Figure 7:**
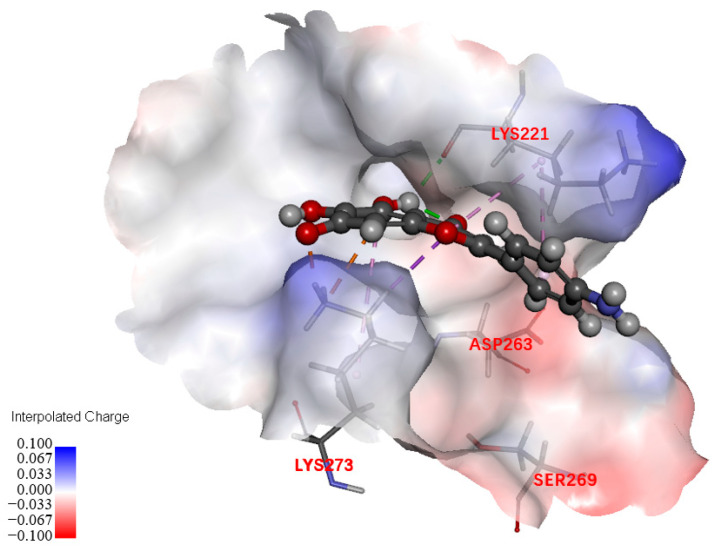
Potential diagram of the combined pocket between **B5** and Eno1.

**Figure 8:**
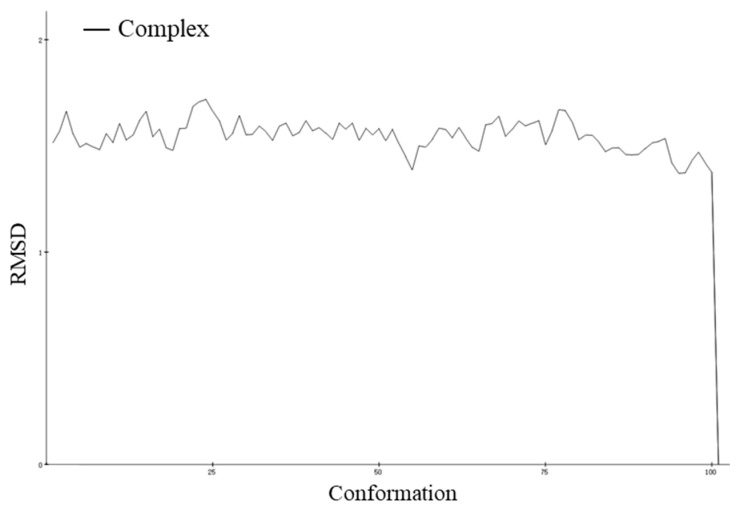
RMSD of different conformations between **B5** and Eno1 complexes.

**Figure 9:**
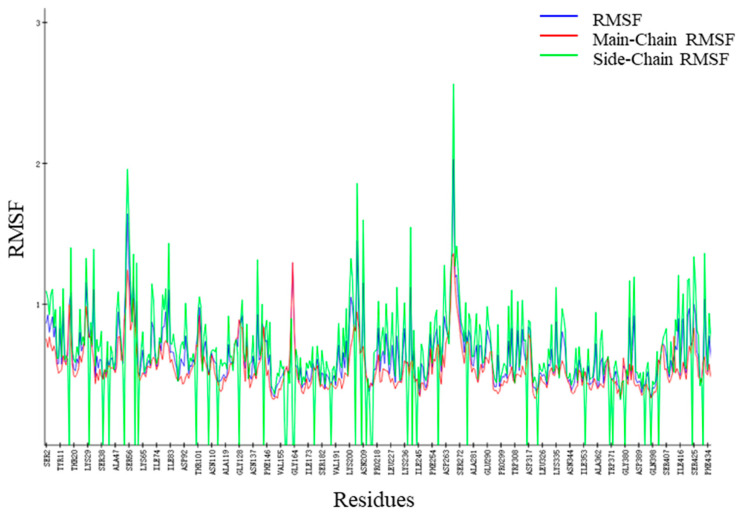
Results of amino acid residue analysis between **B5** and Eno1 complexes.

To understand the reason for the limited affinity of potent compounds binding to Eno1, we chose compound **B5** to perform Molecular Dynamics Simulations (MDs) in conjunction with CaEno1 (PDB ID: 7V67) by virtual docking. Figure 6, Figure 7, Figure 8 and Figure 9 are presented for the obtained results. Comparison of **BE** with compound **B5** indicated that there were distinctive characteristics of hydrogen bond formation between the complex and **B5**. Specifically, the interaction between 5-OH and LYS221 formed a hydrogen bond, while 7-OH formed an ionic bond with LYS273 in **B5** complex rather than a hydrogen bond in **BE** complex. Notably, the absence of a hydrogen bond between 6-OH and SER269 was observed in Figure 6, which diminished the stability of the B5 complex. Examination of the binding pocket potential diagram revealed electrostatic repulsion between the 4′-NH_2_ group of **B5** and LYS273, LYS221. Consequently, the B ring of **B5** could not fit within the cavity formed by LYS273 and LYS221 (Figure 7). Moreover, due to steric hindrance, the anticipated hydrogen bond formation between the 5,6,7-trihydroxy group and SER269, as well as ASP263, was disrupted in comparison to **BE** complex. Thus, the number of hydrogen bonds decreased from three in **BE** complex to one in **B5** complex, potentially decreasing the capacity for enzyme inhibition of **B5**.

**Figure 10:**
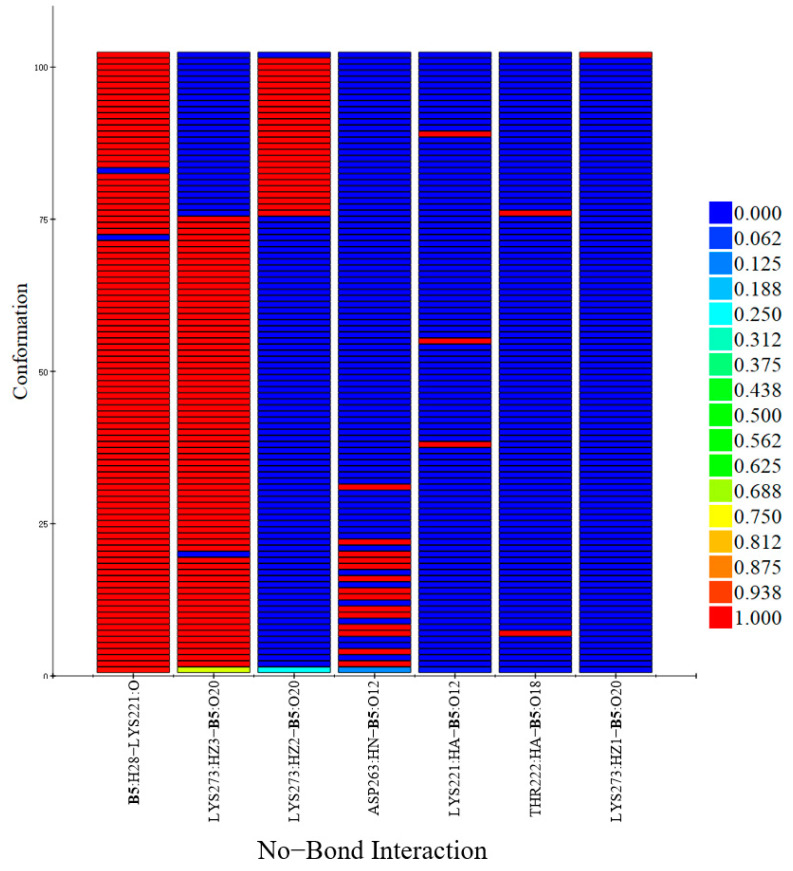
Heat map of hydrogen bond formation in different conformations between **B5** and Eno1 complexes.

During MDs, the RMSD values of the complexes exhibited fluctuations ranging from 1.38614 (conformation 55) to 1.71876 (conformation 24), as depicted in Figure 8, with a variation range of 0.33 Å. The average RMSD value was 1.54786. These outcomes suggested that the complexes remained relatively stable during the simulation, which aligned with the fact that **B5** possessed weaker enzyme inhibitory activity. To investigate the variation of amino acids within the complexes, the RMSF values for all amino acids were calculated (Figure 9). Notably, LYS 55 (RMSF 1.63966), GLN 205 (RMSF 1.45114) and GLU 268 (RMSF 2.03006) exhibited significant fluctuations, indicating higher flexibility at these positions. However, these positions surrounded key residues ASP 263, SER 269 and LYS 273, which was detrimental for maintaining complex stability. The hydrogen heat map (Figure 10) highlights the hydrogen bond distribution within the conformations. The hydrogen bonds between LYS 221-**B5** and LYS 273-**B5** were observed in nearly all conformations, signifying their persistence and stability. This finding was consistent with the docking results. These findings suggest that the enzyme inhibitory ability of **B5** may be compromised due to the reduction in the number of hydrogen bonds. However, the presence of residual hydrogen bonds helped to maintain the stability of **B5** complex. As a result, **B5** exhibited some inhibitory ability in in vitro enzyme inhibition assay, although it was weaker than **BE**.

#### 2.7.2. Exploration of Possible Targets

These findings of the in-silico analysis suggest that our derivatives may influence multiple targets. Subsequently, we utilized Induced-Fit Docking (IFD) to screen proteins that have been reported to be closely associated with hypha and possess potential antifungal effects. The results were further assessed by MDs, and the outcomes are summarized in Table 5. The identified proteins include 1,3-β-d-glucan synthase catalytic subunit [25,26], 1,3-β-d-glucan-UDP glucosyltransferase [27,28], chitin synthase [29,30,31,32], δ-sterol 5-desaturase [33], 14-α demethylase [34], glycosyl-phosphatidylinositol protein [35,36,37], and agglutinin-like protein 3 [12,38].

The binding free energy of enzyme protein receptor and ligand small molecule complexes is calculated by Molecular Mechanics/Generalized Born Surface Area (mmGBSA) [39]. This algorithm calculates the average binding free energy by extracting the architecture of a certain time interval from the MDs’ trajectory of the complex and solving complex interactions between complex molecules by decomposing and calculating the parts that constitute the binding free energy. Then, the obtained free binding energy can be used to react to the stability of the complexes.

**Figure 11:**
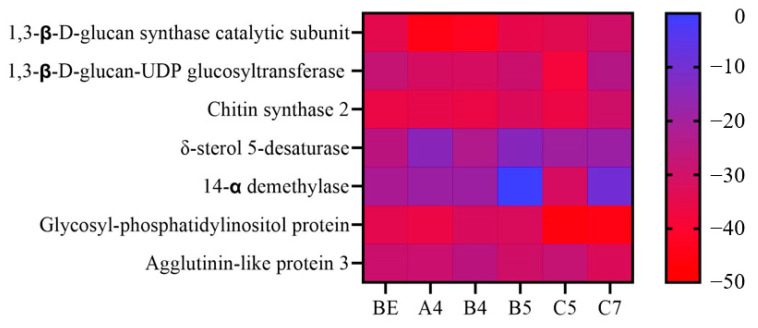
Binding free energy heat map between **BE**, **A4**, **B4**, **B5**, **C5**, **C7** and targets docking results.

The MD results were presented in the form of a heat map (Figure 11), with the values indicating the binding free energy calculated by mmGBSA. Red color represents lower binding free energy, indicating a more stable binding state of the complex. It can be observed that the compounds exhibit greater sensitivity towards the 1,3-β-d-glucan synthase catalytic subunit, 1,3-β-d-glucan-UDP glucosyltransferase, and glycosyl-phosphatidylinositol protein. On the other hand, the interaction between the compounds and sterol biosynthesis pathway related proteins δ-sterol 5-desaturase and 14-α demethylase were weaker. These results are in line with the result of the in vitro sterol composition assay. It could be concluded that these compounds can potentially act on multiple targets.

## 3. Discussion

*C. albicans* is a predominant fungal pathogen, which poses a significant threat to individuals with compromised immune systems, including those with AIDS, tumors, leukemia, and undergoing organ transplants. The mortality rate associated with invasive candidiasis exceeds 40%. Moreover, the emergence of azoles and echinocandins-resistant strains has further complicated antifungal interventions. Consequently, there is an urgent imperative to develop highly effective and minimally toxic antifungal medications and therapies [40,41]. Exploring novel structurally based antifungal agents derived from natural sources represents a crucial avenue of research in this regard.

Several flavonoids have been reported to have antifungal activity, and most of these compounds are characterized by a polyhydroxy structure [42]. However, there is a lack of systematic studies on the antifungal activity and structure–activity relationships of these compounds. In our previous study, **BE** showed significant antifungal activities [16,43]. Inspired by flavonoids with polyhydroxy structures, such as **BE**, we designed and synthesized a series of baicalein-core derivatives and found that compounds **A4**, **B4**, **B5**, **C5** and **C7** exhibited higher synergistic antifungal activity than **BE** when used in combination with FLC. Among these compounds, **A4** and **B5** show the most potent synergistic antifungal activity with MIC_80_ = 0.125 μg/mL (FICI = 0.0088). The SAR indicates that 5-OH is vital to the synergistic antifungal activity, and 3-OH improved this effect. Besides, *o*-dihydroxyls and *vic*-trihydroxy are an essential pharmacophore, either on the A ring or the B ring of these derivatives.

However, the relevant mechanisms are still unknown. Our previous work showed that **BE** displayed potent antifungal effect via hypha formation inhibition and glycolysis disruption [16,18]. Hypha is a structural component of *C. albicans*’ biofilm and plays a crucial role in its development and maintenance. Transitioning from yeast to the hypha morphotype and forming a biofilm greatly contributes to *C. albicans*’ virulence. This impairs the eradication of immune cells and antifungal drugs, allowing *C. albicans* to withstand host immune defenses and increase resistance to antifungal drugs [13]. This process is believed to be necessary for immune cells to escape through the induction of programmed cell death [44]. To investigate the MoA, we initially conducted an in vitro hypha inhibition assay. Compounds **A4**, **B4**, and **B5** all exhibited significant inhibition on formation and quantity of fungal hypha when used in combination with FLC. 

Ergosterol, as the primary sterol in yeast, plays an essential role in maintaining the integrity and function of the cell membrane [45]. The target of azole antifungal drugs is CYP51, which is encoded by the ERG11 gene in fungi. Azoles exert their antifungal activity by blocking ergosterol biosynthesis and causing the accumulation of toxic sterol intermediates [1,46]. Some flavonoids, including Lichochalcone-A [47], 7,4′-dimethylapigenin [48], fisetin [49] and 5,6,8-trihydroxy-7,4′ dimethoxy flavone [50], have an inhibitory effect on fungal ergosterol biosynthesis at the concentration of >51 μg/mL. Thus, the most potent compound **A4** was tested for its effects on the in vitro fungal sterol composition. The results indicated that the combination of **A4** and FLC did not result in significant alterations in the sterol biosynthesis pathway when compared to the use of FLC alone. 

Eno1, encoded by the ENO1 gene, is vital for the survival, pathogenicity, and susceptibility of *C. albicans* to antifungal drugs. Eno1 exhibits enolase activity and is crucial for glycolysis in *C. albicans*. Absence of the ENO1 gene renders *C. albicans* unable to survive on glucose-based media. Additionally, deleting the ENO1 gene increases the susceptibility of *C. albicans* to frontline antifungal drugs, such as amphotericin B, FLC, miconazole, and voriconazole. Therefore, targeting Eno1 shows potential in treating *C. albicans* infections [51,52,53]. **BE** may exert its antifungal effects through inhibition of Eno1 activity [18]. Meanwhile, Mass Spectrometry-Based Proteomic and Immunoproteomic Analyses demonstrate that Eno1 is highly expressed in fungal hypha and secreted through hyphal secretomes. In addition to its glycolytic function, Eno1 can help pathogens evade host immune response [54,55]. Thus, we subjected the compounds to in vitro enzymatic assay with Eno1. Surprisingly, compounds **A4**, **B4**, **B5**, **C5**, and **C7** exhibited weaker Eno1 enzyme inhibition activity compared to **BE**, despite their enhanced antifungal activity when compared to **BE**. 

Therefore, we further used the computer-aided techniques IFD and MD to analyze the possible mechanisms of this class of compounds [56,57,58,59]. Firstly, we found that the changes on the B-ring may lead to a decrease in the number of hydrogen bonds to the complex with Eno1 and compound **B5**, which reduced the inhibitory ability on Eno1. Meanwhile, the amino acid residues around the key residues ASP263, SER269, and LYS273 were more flexible, which was not conducive to the stability of the complex. These may contribute to the fact that **B5** possessed lower enzyme-inhibiting ability than **BE**. These results imply that there may be other targets for this class of compounds, other than just Eno1. Therefore, we further investigated the potential mechanisms of this class of compounds. Our findings revealed that these compounds exhibited greater selectivity for the 1,3-β-d-glucan synthase catalytic subunit, 1,3-β-d-glucan-UDP glucosyltransferase, and glycosyl-phosphatidylinositol protein, as evidenced by IFD and MD analysis. In addition, the results of the IFD and MD analysis also indicated weak binding with sterol biosynthesis pathway related proteins δ-sterol 5-desaturase and 14-α demethylase, which aligned with the results from the in vitro sterol composition assay. 

In addition, Lv’s work shows that **BE** enhances the oxidative stress effect of *C. albicans* by upregulating the expression of CPD2, thereby triggering oxidative damage and apoptosis in fungus [60]. These results also suggest that there could be multiple targets for **BE**. Related studies are still in progress.

## 4. Materials and Methods

### 4.1. Chemistry

Reagents were purchased from common commercial suppliers and were used without further purification. Analytical TLC was carried out on silica gel F254 precoated (0.2 mm thickness) plastic TLC sheets. The TLC plates were spotted with samples using a fine glass capillary tube and developed in a chromatographic tank saturated with solvent vapor at room temperature. ^1^H NMR and ^13^C NMR spectra were recorded on a Bruker Ac 300 or 600 MHz spectrometer in DMSO-*d*_6_ or CDCl_3_ solution. Chemical shifts were recorded in *δ* ppm relative to internal TMS. Mass spectra were obtained by electron spray ionization (ESI) in positive or negative mode using an Aglilent LC/MS-6210 spectrometer. Melting points were determined with an electrothermal melting point apparatus (SGW^®^X-4, Shanghai Shenguang Optical Instrument Factory, Shanghai, China) and were uncorrected.

#### Synthetic Methods for Title Compounds

**6-hydroxy-2-phenyl-4*H*-chromen-4-one (A2)**
  To a solution of MeOH (20 mL), 2′-hydroxy-4′-methoxyacetophenone (185 mg, 1.11 mmol) and benzaldehyde (141.2 mg, 1.33 mmol) were added. Then, 40% NaOH aqueous solution was added dropwise. The mixture was kept at room temperature along with stirring for 24 h. Upon completion of the reaction (monitored by TLC), 50 mL of water were added into the reaction mixture. Then, 2 mol/L diluted hydrochloric acid were added to the mixture to adjust the pH value of the solution to pH 5–6, and the yellow solid precipitate was collected by filtration. After recrystallization with ethyl alcohol, 193 mg of pure white products of 2′-hydroxy-4′-methoxychalcone were obtained with a yield of 60%.
  To a solution of DMSO (20 mL), 2′-hydroxy-4′-methoxychalcone (127 mg, 0.5 mmol) and I_2_ (253 mg, 0.1 mmol) were added, then the mixture was heated to 120–140 °C and kept for 4–8 h. Upon completion of the reaction (monitored by TLC), the reaction mixture was cooled. After 100 mL of water and 100 mg Na_2_S_2_O_3_ were added into the reaction mixture, the yellow solid precipitate was collected by filtration. After recrystallization with ethyl alcohol, 73 mg of pure yellow products of 6-methoxyflavone (**A23**) were obtained with a yield of 59%.
  To a solution of anhydrous DCM (10 mL), **A23** (71 mg, 0.28 mmol) and 1 M of BBr_3_/DCM solution (2.8 mL, 1.4 mmol) were added at a temperature of −15 °C under argon atmosphere condition. Then the mixture was kept, along with stirring at room temperature, for another 12 h. After quenching the reaction by adding 10 mL of water at the temperature of −15 °C, the mixture was poured into a separatory funnel and separated. The aqueous layer was extracted with DCM (30 mL) three times. The combined organic layers were washed with saturated NaCl solution and dried over anhydrous Na_2_SO_4_ and evaporated to dryness to give crude products. After recrystallization with ethyl alcohol, 50 mg of pure yellow products of 6-hydroxyflavone (**A2**) were obtained with a yield of 42%.
  ^1^H NMR (300 MHz, DMSO-*d*_6_) δ 10.04 (s, 1H), 8.12–8.03 (m, 2H), 7.66 (d, *J* = 9.0 Hz, 1H), 7.66–7.51 (m, 3H), 7.33 (d, *J* = 2.9 Hz, 1H), 7.26 (dd, *J* = 9.0, 3.0 Hz, 1H), 6.96 (s, 1H). ^13^C NMR (75 MHz, DMSO-*d*_6_) δ 177.00, 162.18, 154.90, 149.39, 131.62, 131.38, 129.09 (×2), 126.24 (×2), 124.24, 123.10, 119.85, 107.49, 105.93.

**5,6-dihydroxy-2-phenyl-4*H*-chromen-4-one (A4)**
  **A2** was oxidized by iodo-benzene diacetate (IBD) according to the reference method to give **A14** [20], then demethylated by BBr_3_ to **A4**.
  ^1^H NMR (300 MHz, DMSO-d6) δ 10.04 (s, 1H), 8.12–8.03 (m, 2H), 7.66 (d, *J* = 9.0 Hz, 1H), 7.66–7.51 (m, 3H), 7.33 (d, *J* = 2.9 Hz, 1H), 7.26 (dd, *J* = 9.0, 3.0 Hz, 1H), 6.96 (s, 1H).^13^C NMR (75 MHz, DMSO) δ 177.00, 162.18, 154.90, 149.39, 131.62, 131.38, 129.09 (×2), 126.24 (×2), 124.24, 123.10, 119.85, 107.49, 105.93.

**6,7-dihydroxy-2-phenyl-4*H*-chromen-4-one (A6)**
  2′-hydroxy-3′,4′-methyleneacetophenone was reacted with benzaldehyde in a similar way as described for **A2** to give the product **A6**.
  ^1^H NMR (600 MHz, DMSO-*d*_6_) δ 10.45 (s, 1H), 9.79 (s, 1H), 8.05–8.00 (m, 2H), 7.61–7.51 (m, 3H), 7.31 (s, 1H), 7.04 (s, 1H), 6.83 (s, 1H). ^13^C NMR (151 MHz, DMSO-*d*_6_) δ 176.22, 161.43, 152.41, 150.86, 144.69, 131.60, 131.29, 129.05 (×2), 126.02 (×2), 116.16, 107.62, 105.97, 103.20.

**6,7-dimethoxy-2-phenyl-4*H*-chromen-4-one (A11)**
  Dissolve 1 equivalent of **A6** in THF, add 2 equivalents of K_2_CO_3_ and CH_3_I, reflux for 8 h at 70 °C, stop heating after monitoring the reaction completely by TLC, and cool to room temperature. The mixture was poured into 100mL of water and stirred for 30 min and filtered by extraction. The filter cake was oven dried and then recrystallized in methanol to obtain **A11**.
  ^1^H NMR (300 MHz, Chloroform-*d*) δ 7.95–7.85 (m, 2H), 7.55 (s, 1H), 7.50 (dd, *J* = 5.2, 1.9 Hz, 3H), 6.99 (d, *J* = 1.5 Hz, 1H), 6.83 (d, *J* = 1.5 Hz, 1H), 4.01 (s, 3H), 3.97 (d, *J* = 1.7 Hz, 3H).
  ^13^C NMR (75 MHz, CDCl_3_) δ 177.84, 162.91, 154.60, 152.38, 147.74, 131.88, 131.37, 129.02 (×2), 126.10 (×2), 117.24, 106.98, 104.37, 99.77, 56.50, 56.37.

**9-hydroxy-6-phenyl-8*H*-[1,3]dioxolo[4,5-*g*]chromen-8-one (A13)**
  A suspension of baicalein (100 mg, 0.37 mmol), BrCH_2_Cl (52.1 mg, 0.41 mmol) in ethanol (15 mL) and Cs_2_CO_3_ (100 mg) was heated to reflux with stirring at Ar_2_ atmosphere for 12 h. Then the mixture was poured into ice water. The solid precipitate was collected by filtration. The crude product was purified by silica gel chromatography eluted with PE: EtOAc = 25:1 to give product (80 mg, 77%) as yellow solid. M^+^ = 283.0, M + Na^+^ = 305.0.
  ^1^H NMR (300 MHz, Chloroform-*d*) δ 12.70 (s, 1H), 7.86 (dt, *J* = 7.6, 1.4 Hz, 2H), 7.61–7.45 (m, 3H), 6.67 (s, 1H), 6.59 (s, 1H), 6.10 (s, 2H).^13^C NMR (75 MHz, CDCl_3_) δ 183.15, 164.16, 154.24, 153.43, 142.36, 132.01, 131.29, 130.26, 129.25 (×2), 126.37 (×2), 107.91, 105.66, 102.82, 89.62.

**6-hydroxy-5-methoxy-2-phenyl-4*H*-chromen-4-one (A14)**
  A suspension of 6-hydroxyflavone (100 mg, 0.66 mmol), IBD (278 mg, 0.77 mmol) in methanol (15 mL), was kept at room temperature along with stirring for 2 h, then heated to reflux for 1 h. After removal of solvent by under reduced pressure, the residues were purified by silica gel chromatography eluted with PE: EtOAc = 20:1 to give a pure yellow solid (96 mg, 54%). M^+^ = 269.3.
  ^1^H NMR (300 MHz, DMSO-*d*_6)_ δ 8.02 (dd, *J* = 8.3, 1.5 Hz, 2H), 7.69–7.49 (m, 4H), 6.60 (s, 1H), 6.52 (dd, J = 10.3, 2.0 Hz, 1H), 3.43 (s, 3H). ^13^C NMR (75 MHz, DMSO-*d*_6_) δ 184.93, 181.04, 165.44, 144.04, 140.40, 132.58, 131.25, 130.21, 129.07 (×2), 126.83, 126.71 (×2), 103.07, 95.62, 52.02.

**5-methoxy-2-phenyl-4*H*-chromen-4-one (A17)**
  2′-hydroxy-6′-methoxyacetophenone was reacted with benzaldehyde in a similar way as described for **A2** to give the product **A17**.
  ^1^H NMR (300 MHz, Chloroform-*d*) δ 7.87 (dd, *J* = 6.9, 2.9 Hz, 2H), 7.55 (t, *J* = 8.4 Hz, 1H), 7.48 (dd, *J* = 5.2, 1.9 Hz, 3H), 7.11 (d, *J* = 8.4 Hz, 1H), 6.81 (d, *J* = 8.3 Hz, 1H), 6.72 (s, 1H), 3.98 (s, 3H). ^13^C NMR (75 MHz, Chloroform-*d*) δ 178.38, 161.13, 159.83, 158.35, 133.82, 131.50, 131.41, 129.02, 126.11, 114.66, 110.22, 109.14, 106.53, 56.57.

**6,8-dibromo-5,7-dihydroxy-2-phenyl-4*H*-chromen-4-one (A18)**
  To a suspension of AcOH (15 mL) and chrysin (**A5**, 254 mg, 1 mmol), Br_2_ (3 mmol) was added, along with stirring at room temperature. Then the mixture was kept for 3 h. Upon completion of the reaction (monitored by TLC), 100 mL of water and 100 mg Na_2_S_2_O_3_ were added into the reaction mixture, and the yellow solid precipitate was collected by filtration. After recrystallization with EtOH, pure product was obtained (336 mg, 82%). M^+^ = 411.1, 413.2.
  ^1^H NMR (300 MHz, DMSO-*d*_6_) δ 13.73 (s, 1H), 8.17–8.09 (m, 2H), 7.61 (d, *J* = 7.3 Hz, 3H), 7.19 (s, 1H). ^13^C NMR (75 MHz, DMSO-*d*_6_) δ 181.56, 163.48, 157.50, 157.03, 152.30, 132.48, 130.24, 129.26 (×2), 126.47 (×2), 105.13, 105.04, 94.58, 88.48.

**5,7-dihydroxy-8-nitro-2-phenyl-4*H*-chromen-4-one (A19)**
  A19 was synthesized according to the references with 62% yield [21]. ^1^H NMR (300 MHz, DMSO-*d*_6_) δ 6.37 (s, 1H), 7.19 (s, 1H), 7.61 (t, *J* = 6.6 Hz, 3H), 7.91–8.06 (m, 2H), 13.23 (s, 1H).
  ^1^H NMR (300 MHz, DMSO-*d*_6_) δ 13.24 (s, 1H), 8.03–7.94 (m, 2H), 7.70–7.54 (m, 3H), 7.20 (s, 1H), 6.38 (s, 1H).

**5,7-dimethoxy-8-nitro-2-phenyl-4*H*-chromen-4-one (A20)**
  **A19** was reacted with CH_3_I in a similar way as described for **A11** to give the product **A20**.
  ^1^H NMR (300 MHz, Chloroform-*d*) δ 7.84–7.75 (m, 2H), 7.49 (d, *J* = 7.1 Hz, 3H), 6.72 (s, 1H), 6.43 (s, 1H), 4.07 (s, 3H), 4.06 (s, 3H). ^13^C NMR (75 MHz, Chloroform-*d*) δ 176.14, 162.52, 161.01, 156.16, 151.16, 132.03, 130.38, 129.30 (×2), 126.31 (×2), 109.03, 108.33, 91.36, 77.36, 57.04 (×2).

**5,6,7-trimethoxy-2-phenyl-4*H*-chromen-4-one (A21)**
  **BE** was reacted with CH_3_I in a similar way as described for **A11** to give the product **A21**.
  ^1^H NMR (300 MHz, Chloroform-*d*) δ 7.93–7.82 (m, 2H), 7.50 (dd, *J* = 5.1, 1.9 Hz, 3H), 6.81 (s, 1H), 6.67 (s, 1H), 3.98 (s, 3H), 3.98 (s, 3H), 3.91 (s, 3H). 13C NMR (75 MHz, Chloroform-*d*) δ 177.32, 161.25, 157.93, 154.70, 152.69, 140.55, 131.71, 131.39, 129.09 (×2), 126.09 (×2), 113.08, 108.52, 96.41, 62.31, 61.66, 56.43.

**8-amino-5-hydroxy-7-methoxy-2-phenyl-4*H*-chromen-4-one (A22)**
  **A19** was reacted with equimolar CH_3_I in a similar way as described for **A11** to give the 5-hydroxy-7-methoxy-8-nitroflavone. 5-hydroxy-7-methoxy-8-nitroflavone was reduced in a similar way as described for **A19** to give the **A22**.
  ^1^H NMR (300 MHz, Chloroform-*d*) δ 12.10 (s, 1H), 7.92–7.80 (m, 2H), 7.53 (q, *J* = 4.1 Hz, 3H), 6.60 (s, 1H), 6.42 (s, 1H), 3.93 (s, 3H). ^13^C NMR (75 MHz, Chloroform-*d*) δ 183.04, 163.75, 153.73, 153.02, 143.86, 131.89, 131.80, 129.23 (×2), 126.40 (×2), 116.05, 105.66, 104.99, 95.32, 56.29.

**6-methoxy-2-phenyl-4*H*-chromen-4-one (A23)**
  1-(2-hydroxy-5-methoxyphenyl) ethan-1-one was reacted with benzaldehyde in a similar way as described for **A2** to give the product **A23**.
  ^1^H NMR (300 MHz, Chloroform-*d*) δ 7.98–7.88 (m, 2H), 7.64–7.45 (m, 3H), 7.44 (d, *J* = 2.3 Hz, 1H), 7.34 (d, *J* = 2.7 Hz, 1H), 7.13–7.02 (m, 1H), 6.83 (s, 1H), 3.91 (s, 3H).
  ^13^C NMR (75 MHz, CDCl_3_) δ 178.20, 166.62, 157.41, 151.16, 131.63, 130.49, 129.17 (×2), 126.39 (×2), 125.52, 123.95, 119.93, 105.19, 105.02, 90.58, 56.11.

**6-phenyl-8*H*-[1,3]dioxolo[4,5-*g*] chromen-8-one (A24)**
  2′-hydroxy-3′,4′-methyleneacetophenone was reacted with benzaldehyde in a similar way as described for **A2** to give the product **A24**.
  ^1^H NMR (600 MHz, DMSO-*d*_6_) δ 8.07–8.02 (m, 2H), 7.61–7.50 (m, 3H), 7.37 (d, *J* = 0.8 Hz, 1H), 7.32 (d, *J* = 0.7 Hz, 1H), 6.95 (d, *J* = 0.7 Hz, 1H), 6.21 (s, 2H). ^13^C NMR (151 MHz, DMSO) δ 175.97, 161.79, 152.93, 152.60, 146.05, 131.53, 131.09, 129.04 (×2), 126.05 (×2), 118.18, 106.22, 102.80, 100.82, 98.48.

**5,6,7-trimethoxy-2-(4-nitrophenyl)-4*H*-chromen-4-one (B1)**
  1-(6-hydroxy-2,3,4-trimethoxyphenyl) ethan-1-one was reacted with 4-nitrobenzaldehyde in a similar way as described for **A2** to give the product **B1**.
  ^1^H NMR (300 MHz, DMSO-*d*_6_) δ 8.36 (s, 4H), 7.27 (s, 1H), 7.03 (s, 1H), 3.96 (s, 3H), 3.81 (s, 3H), 3.78 (s, 3H).

**5,6,7-trihydroxy-2-(4-nitrophenyl)-4*H*-chromen-4-one (B2)**
  **B1** was reacted in a similar way as described for **A2** to give the product **B2**.
  ^1^H NMR (300 MHz, DMSO-*d*_6_) δ 12.53 (s, 1H), 10.68 (s, 1H), 8.33 (t, *J* = 3.3 Hz, 4H), 7.11 (s, 1H), 6.64 (s, 1H). ^13^C NMR (75 MHz, DMSO-*d*_6_) δ 181.91, 160.32, 153.99, 149.83, 148.99, 146.87, 136.87, 129.58, 127.68 (×2), 124.05 (×2), 106.81, 104.51, 94.15.

**2-(4-bromophenyl)-5,6,7-trimethoxy-4*H*-chromen-4-one (B3)**
  1-(6-hydroxy-2,3,4-trimethoxyphenyl) ethan-1-one was reacted with 4-bromobenzaldehyde in a similar way as described for **A2** to give the product **B3**.
  ^1^H NMR (300 MHz, Chloroform-*d*) δ 7.74 (d, *J* = 8.4 Hz, 2H), 7.64 (d, *J* = 8.3 Hz, 2H), 6.80 (s, 1H), 6.67 (d, *J* = 1.2 Hz, 1H), 3.98 (d, *J* = 1.2 Hz, 6H), 3.92 (s, 3H).

**2-(4-bromophenyl)-5,6,7-trihydroxy-4*H*-chromen-4-one (B4)**
  **B3** was reacted in a similar way as described for **A2** to give the product **B4**.
  ^1^H NMR (600 MHz, DMSO-*d*_6_) δ 12.58 (s, 1H), 10.59 (s, 1H), 7.99 (d, *J* = 8.4 Hz, 2H), 7.75 (d, *J* = 8.3 Hz, 2H), 6.95 (s, 1H), 6.61 (s, 1H).

**2-(4-aminophenyl)-5,6,7-trihydroxy-4*H*-chromen-4-one (B5)**
  **B2** was reduced in a similar way as described for **A19** to give the **B5**.
  ^1^H NMR (300 MHz, DMSO-*d*_6_) δ 12.97 (s, 1H), 10.38 (s, 1H), 8.67 (s, 1H), 7.74 (d, *J* = 8.5 Hz, 2H), 6.65 (d, *J* = 8.5 Hz, 2H), 6.59 (s, 1H), 6.53 (s, 1H), 6.04 (s, 2H). ^13^C NMR (75 MHz, DMSO-*d*_6_) δ 181.78, 164.37, 152.95, 152.75, 149.51, 147.15, 128.94, 128.01 (×2), 116.82, 113.46 (×2), 103.83, 100.25, 93.69.

**5,6,7-trimethoxy-2-(3-nitrophenyl)-4*H*-chromen-4-one (B6)**
  1-(6-hydroxy-2,3,4-trimethoxyphenyl) ethan-1-one was reacted with 3-nitrobenzaldehyde in a similar way as described for **A2** to give the product **B6**.
  ^1^H NMR (300 MHz, DMSO-*d*_6_) δ 8.81 (s, 1H), 8.52 (s, 1H), 8.41 (s, 1H), 7.83 (d, *J* = 7.2 Hz, 1H), 7.31 (s, 1H), 7.03 (s, 1H), 3.97 (s, 3H), 3.81 (s, 3H), 3.77 (s, 3H).

**5,6,7-trihydroxy-2-(3-nitrophenyl)-4*H*-chromen-4-one (B7)**
  **B6** was reacted in a similar way as described for **A2** to give the product **B7**.
  ^1^H NMR (300 MHz, DMSO-*d*_6_) δ 12.51 (s, 1H), 10.65 (s, 1H), 8.75 (t, *J* = 2.0 Hz, 1H), 8.53–8.44 (m, 1H), 8.40 (dd, *J* = 8.1, 2.3 Hz, 1H), 7.84 (t, *J* = 8.1 Hz, 1H), 7.13 (s, 1H), 6.66 (s, 1H). ^13^C NMR (75 MHz, DMSO-*d*_6_) δ 182.01, 160.37, 153.90, 149.79, 148.38, 146.90, 132.71, 132.55, 130.78, 129.53, 126.05, 120.84, 106.06, 104.42, 94.19.

**2-(3-aminophenyl)-5,6,7-trihydroxy-4*H*-chromen-4-one (B8)**
  **B7** was reduced in a similar way as described for **A19** to give the **B8**.
  ^1^H NMR (300 MHz, DMSO-*d*_6_) δ 12.65 (s, 1H), 7.16 (dt, *J* = 9.6, 4.8 Hz, 3H), 6.76 (d, *J* = 7.7 Hz, 1H), 6.67 (s, 1H), 6.55 (s, 1H), 5.41 (s, 2H). ^13^C NMR (75 MHz, DMSO-*d*_6_) δ 181.95, 163.82, 149.88, 149.32, 146.79, 131.54, 129.65 (×2), 129.40, 117.21, 113.73, 110.79, 104.05, 103.94 (×2), 93.77.

**2-(3,4-dihydroxyphenyl)-5-hydroxy-4*H*-chromen-4-one (B10)**
  1-(2-hydroxy-6-methoxyphenyl) ethan-1-one was reacted with 3,4-dimethoxybenzaldehyde in a similar way as described for **A2** to give the product **B10**.
  ^1^H NMR (300 MHz, DMSO-*d*_6_) δ 12.84 (s, 1H), 9.77 (s, 2H), 7.65 (t, *J* = 8.3 Hz, 1H), 7.53–7.42 (m, 2H), 7.13 (dd, *J* = 8.4, 0.9 Hz, 1H), 6.91 (d, *J* = 8.2 Hz, 1H), 6.83 (s, 1H), 6.82–6.76 (m, 1H). ^13^C NMR (75 MHz, DMSO-*d*_6_) δ 182.85, 164.85, 159.90, 155.79, 150.08, 145.81, 135.66, 121.30, 119.35, 116.04, 113.59, 110.84, 109.93, 107.26, 103.40.

**2-(benzo[*d*][1,3]dioxol-5-yl)-5-methoxy-4*H*-chromen-4-one (B14)**
  2′-hydroxy-6′-methoxyacetophenone was reacted with benzo[*d*][1,3]dioxole-5-carbaldehyde in a similar way as described for **A2** to give the product **B14**.
  ^1^H NMR (300 MHz, DMSO-*d*_6_) δ 7.68 (t, *J* = 8.4 Hz, 1H), 7.63 (dt, *J* = 4.4, 2.2 Hz, 2H), 7.27 (dd, *J* = 8.4, 0.9 Hz, 1H), 7.09 (d, *J* = 8.7 Hz, 1H), 6.98 (d, *J* = 8.3 Hz, 1H), 6.78 (s, 1H), 6.15 (s, 2H), 3.86 (s, 3H). ^13^C NMR (75 MHz, DMSO-*d*_6_) δ 176.42, 159.74, 159.03, 157.48, 150.13, 148.15, 134.14, 124.68, 121.17, 113.64, 110.00, 108.68, 107.37, 107.20, 106.09, 101.95, 56.09.

**5-methoxy-2-(thiophen-2-yl)-4*H*-chromen-4-one (B15)**
  2′-hydroxy-6′-methoxyacetophenone was reacted with thiophene-2-carbaldehyde in a similar way as described for **A2** to give the product **B15**.
  ^1^H NMR (300 MHz, DMSO-*d*_6_) δ 7.95 (dd, *J* = 3.8, 1.2 Hz, 1H), 7.93 (dd, *J* = 5.0, 1.2 Hz, 1H), 7.66 (t, *J* = 8.4 Hz, 1H), 7.26 (dd, *J* = 5.0, 3.8 Hz, 1H), 7.15 (dd, *J* = 8.4, 0.9 Hz, 1H), 6.97 (dd, *J* = 8.5, 1.0 Hz, 1H), 6.71 (s, 1H), 3.85 (s, 3H). ^13^C NMR (75 MHz, DMSO-*d*_6_) δ 175.88, 159.10, 157.20, 156.24, 134.29, 133.83, 131.29, 129.08, 128.89, 113.69, 109.72, 107.44, 106.80, 56.11.

**2-(furan-2-yl)-5-methoxy-4*H*-chromen-4-one (B16)**
  2′-hydroxy-6′-methoxyacetophenone was reacted with furan-2-carbaldehyde in a similar way as described for **A2** to give the product **B16**.
  ^1^H NMR (300 MHz, Chloroform-*d*) δ 7.56 (dd, *J* = 7.3, 1.1 Hz, 1H), 7.51 (dd, *J* = 8.4, 1.0 Hz, 1H), 7.08–6.99 (m, 2H), 6.79 (d, *J* = 8.3 Hz, 1H), 6.61 (s, 1H), 6.56 (dd, *J* = 3.5, 1.6 Hz, 1H), 3.96 (s, 3H). ^13^C NMR (75 MHz, Chloroform-*d*) δ 177.83, 159.86, 157.92, 153.26, 146.21, 145.62, 133.82, 114.84, 112.65, 112.45, 110.09, 107.16, 106.66, 56.57.

**5-hydroxy-2-(thiophen-2-yl)-4*H*-chromen-4-one (B17)**
  The 2′-hydroxy-6′-methoxyacetophenone was reacted with thiophene-2-carbaldehyde in a similar way as described for **A2** to give the product **B17**.
  ^1^H NMR (300 MHz, Methanol-*d*_4_) δ 7.79 (d, *J* = 3.8 Hz, 1H), 7.67 (d, *J* = 4.9 Hz, 1H), 7.61–7.45 (m, 2H), 7.20 (t, *J* = 4.4 Hz, 1H), 6.99 (d, *J* = 8.4 Hz, 1H), 6.77 (d, *J* = 8.2 Hz, 1H), 6.61 (s, 1H). ^13^C NMR (75 MHz, Methanol-*d*_4_) δ 183.87, 161.25, 160.74, 156.64, 136.12, 134.70, 131.98, 130.08, 129.22, 111.98, 111.02, 107.75, 104.55.

### 4.2. Antifungal Activity (Broth Microdilution Method)

The clinical isolates of FLC-resistant *C. albicans* 103, 938 and 939 were used in this study, and *C. albicans* ATCC 90028 was used as a quality control. The antifungal properties of the title compounds were evaluated by the broth microdilution method according to the NCCLS reference document M27-A3 [22]. **BE** was used as positive control. The MIC_80_ of FLC against the FLC-resistant *C. albicans* (clinical isolate 103, 938 and 939) was determined to be 128 μg/mL. The MIC_80_ of each title compound when used alone and when combined with FLC (8.0 μg/mL) are described in Table 1, Table 2 and Table 3. Each compound was tested in triplicate. Furthermore, the fractional inhibitory concentration index (FICI) of each agent was calculated by adding the ratios of the MIC_80_ (with FLC)/MIC_80_ (used alone). The interaction modes—synergistic or indifferent—were defined according to FICI values of ≤0.5 or >0.5, respectively.

### 4.3. In Vitro Hyphal Formation Assay

The clinical isolates of FLC-resistant *C. albicans* 901,904 underwent exponential growth in YEPD medium. The cells were then diluted with 1.5 mL of spider medium to obtain a concentration of 1 × 10^5^ CFU/mL. The fungal cell suspension was subsequently added to a 12-well plate. Compound **A4**, **B4**, **B5** at a concentration of 8 μg/mL, along with FLC as the positive drug, were added. After incubating the fungal cells at 37 °C for 7 h, the morphological differences between the drug-treated group and the group without drugs were recorded using a live cell imaging inverted microscope [61].

### 4.4. Analysis of Fungal Sterol Composition Assay

After treatment with compound **A4** with a concentration of 8 μg/mL in YEPD medium, the *C. albicans* SC5314 cells were collected (wet weight about 1.0 g). Subsequently, the mixtures were combined with 10 mL of saponifier (a solution of 90% ethanol and 15% NaOH) and heated to 80 °C for 1 h. Following this, 10 mL of petroleum ether (with a boiling range of 30–60 °C) was added three times to extract the sterols from each group. The extracted solutions were then evaporated under reduced pressure, and the residue was dissolved in 1 mL of cyclohexane. Finally, the composition of sterols in each compound group was analyzed using GC-MS. To identify the sterols, we matched the molecular fragments from each peak in the GC-MS chromatograms with the corresponding sterol compounds in the NIST (the National Institute of Standards and Technology) reference database [61].

### 4.5. Enolase Activity Analysis

Enolase activity was determined by direct spectrophotometric assay via measuring the increase of absorbance at 240 nm of phosphoenolpyruvate (PEP) as described previously with some modifications [62]. Briefly, the reaction buffer (pH 7.0) containing 10 mM imidazole, 200 mM KCl, and 0.5 mM MgAc in a final reaction volume of 100 mL was mixed with Eno1 (a final concentration of 30 nM), followed by the mixture of 2-phosphoglycerate (2-PG) (Yuanye Biotech Shanghai, China) with a final concentration of 1 mM. The enolase activity of Eno1 was evaluated by measuring the increase of absorbance (OD240) at room temperature for 10 min. For the inhibition study of enolase by compounds, compounds at a concentration of 200 μM were used to mix well with Eno1 and were incubated at room temperature for 5 min, and subsequent operations were described above.

### 4.6. Docking Studies

Docking studies of all baicalein derivatives were performed in the Discovery Studio. The protein crystal structure of CaEno1 (PDB ID: 7V67) was obtained by downloading from the PDB (https://www.rcsb.org/, accessed on 12 January 2023). The protein is prepared using Prepare Protein module, including hydrogenation, removal of water-free molecules, repair of protein side chains and modification of C and N termini, and repair of disordered chemical bonds and atoms. The parameters related to this process are default values. After importing the compound set into DS, these compounds as ligands are prepared using Prepare Ligands module for docking, including changing the ionization state, generating reciprocal isomers and stereoisomers, etc. The parameters related to this procedure are default values. The binding sites were set regarding the literature [18], with key residues ASP 263, SER 269, and LYS 273 set as binding pockets. the radius was set to 15 Å. Semi-flexible docking was performed using CDOCKER, and the parameters were all default values.

Molecular dynamic simulation of complexes was generated by docking of compound B5 using the Standard Dynamic Cascade module. The force field was chosen as charmm36 [63] while solventization was performed afterward. All parameters are at default values. A total of 9795 water molecules, 26 sodium ions, and 26 chloride ions were added. Afterward, the equilibrium phase was set to 20 ps and the runtime phase was set to 200 ps to perform molecular dynamic simulation.

IFD and mmGBSA were carried out by Schrödinger software by using the corresponding module, and proteins 1,3-β-d-glucan synthase catalytic subunit (Protein ID: O13383), 1,3-β-d-glucan-UDP glucosyltransferase (Protein ID: O13428), δ-sterol 5-desaturase (Protein ID: O93875), glycosyl-phosphatidylinositol protein (Protein ID: AAG29538) Agglutinin-like protein 3 (PDB ID: 4LEB) were downloaded from the RCSB Protein Data Bank (https://www.rcsb.org/, accessed on 12 January 2023) library.

## 5. Conclusions

The emergence of drug resistance in fungi is outpacing the development of new anti-fungal treatments. To address this, alternative approaches such as combination therapy, anti-virulence agents, and modulation of host immune responses are being explored [64]. Combination therapy has shown promise in overcoming resistance by targeting multiple mechanisms of action and reducing pathogen populations. It can also reverse drug resistance and unveil new treatment options. Additionally, combination therapy increases the efficiency of drugs and makes it more difficult for pathogens to develop resistance [65,66]. Our **BE**-core derivatives exhibited a strong inhibitory effect on hypha formation, which makes these compounds act as ideal synergistic small molecules, since hypha are one of the important virulence factors in *C. albicans* infections.

SAR study provides *o*-dihydroxyls, and *vic*-trihydroxy are an essential pharmacophore, either on the A ring or the B ring of these derivatives. Analyses of MoA showed that these compounds retained the effect on inhibition of hypha formation. However, there was little effect on ergosterol biosynthesis and Eno1. In silico studies explained that these results were due to the hydrogen bond reduction and key residues instability. Further studies indicated that the 1,3-β-d-glucan synthase catalytic subunit, 1,3-β-d-glucan-UDP glucosyltransferase, and glycosyl-phosphatidylinositol protein could be the candidate targets of these compounds. Taken together, this study suggests some potential for synergistic antifungal agents and an effective strategy for treatment of FLC-resistant *C. albicans* infections.

## Figures and Tables

**Table 1:** Antifungal activity of **A1**–**A25** alone and in combination with FLC against FLC-resistant *C. albicans* 103. (MIC_80_, μg/mL, 48 h).

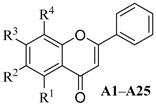								
Compd.	R^1^	R^2^	R^3^	R^4^	Alone	with FLC ^[a]^	FICI	Mode of Interaction
**A1**	OH	H	H	H	>64	4	0.094	Synergism
**A2**	H	OH	H	H	>64	>64	2.000	Indifferent
**A3**	H	H	OH	H	>64	>64	2.000	Indifferent
**A4**	OH	OH	H	H	>64	1	0.070	Synergism
**A5**	OH	H	OH	H	>64	64	0.563	Addition
**A6**	H	OH	OH	H	>64	8	0.125	Synergism
**A7**	H	H	OH	OH	>64	4	0.094	Synergism
**A8**	OH	OH	OCH_3_	H	>64	4	0.094	Synergism
**A9**	OH	OCH_3_	OH	H	>64	4	0.094	Synergism
**A10**	OH	H	OCH_3_	H	>64	16	0.188	Synergism
**A11**	H	OCH_3_	OCH_3_	H	>64	>64	2.000	Indifferent
**A12**	OH	OCH_3_	OCH_3_	H	>64	8	0.125	Synergism
**A13**	OH	-OCH_2_O-		H	>64	4	0.094	Synergism
**A14**	OCH_3_	OH	H	H	>64	32	0.313	Synergism
**A15**	OCH_3_	H	OCH_3_	H	>64	64	0.563	Addition
**A16**	OH	H	OH	OCH_3_	>64	8	0.125	Synergism
**A17**	OCH_3_	H	H	H	>64	64	0.563	Addition
**A18**	OH	Br	OH	Br	>64	4	0.094	Synergism
**A19**	OH	H	OH	NO_2_	>64	16	0.188	Synergism
**A20**	OCH_3_	H	OCH_3_	NO_2_	>64	32	0.313	Synergism
**A21**	OCH_3_	OCH_3_	OCH_3_	H	>64	>64	2.000	Indifferent
**A22**	OH	H	OCH_3_	NH_2_	>64	>64	2.000	Indifferent
**A23**	H	OCH_3_	H	H	>64	>64	2.000	Indifferent
**A24**	H	-OCH_2_O-		H	>64	4	0.094	Synergism
**A25**	OH	OH	*O*-(1-glucuronide)	H	>64	16	0.188	Synergism
**BE**	OH	OH	OH	H	32	8	0.313	Synergism

^[a]^ MIC_80_ value of compound in combination with 8.0 μg/mL FLC.

**Table 2:** Susceptibilities of **B1**–**B17** alone and in combination with FLC against FLC-resistant *C. albicans* 103. (MIC80, μg/mL, 48 h).

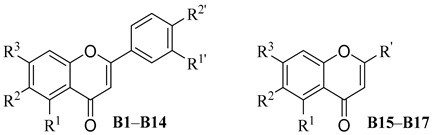										
Compd.	R^1^	R^2^	R^3^	R^1′^	R^2′^	R^’^	Alone	with FLC ^[a]^	FICI	Mode of Interaction
**B1**	OCH_3_	OCH_3_	OCH_3_	H	NO_2_	–	>64	>64	2.000	Indifferent
**B2**	OH	OH	OH	H	NO_2_	–	>64	2	0.078	Synergism
**B3**	OCH_3_	OCH_3_	OCH_3_	H	Br	–	>64	>64	2.000	Indifferent
**B4**	OH	OH	OH	H	Br	–	32	4	0.188	Synergism
**B5**	OH	OH	OH	H	NH_2_	–	16	2	0.188	Synergism
**B6**	OCH_3_	OCH_3_	OCH_3_	NO_2_	H	–	>64	>64	2.000	Indifferent
**B7**	OH	OH	OH	NO_2_	H	–	>64	4	0.094	Synergism
**B8**	OH	OH	OH	NH_2_	H	–	16	4	0.313	Synergism
**B9**	OH	OH	OH	H	OH	–	>64	16	0.188	Synergism
**B10**	OH	H	H	OH	OH	–	>64	32	0.313	Synergism
**B11**	OH	H	OH	OH	OH	–	>64	32	0.313	Synergism
**B12**	OH	H	OH	OH	OCH_3_	–	>64	64	0.563	Addition
**B13**	OH	H	OH	H	OH	–	>64	64	0.563	Addition
**B14**	OCH_3_	H	H	-OCH_2_O-		–	>64	>64	2.000	Indifferent
**B15**	OCH_3_	H	H	–	–	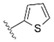	>64	64	0.563	Addition
**B16**	OCH_3_	H	H	–	–	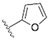	>64	64	0.563	Addition
**B17**	OH	H	H	–	–	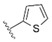	>64	8	0.125	Synergism
**BE**	OH	OH	OH	H	H	–	64.0	4	0.125	Synergism

^[a]^ MIC_80_ value of compound in combination with 8.0 μg/mL FLC. –: no substitution.

**Table 3:** Susceptibilities of **C1**–**C16** and **D1**–**D4** alone and in combination with FLC against FLC-resistant *C. albicans* 103. (MIC80, μg/mL, 48 h).

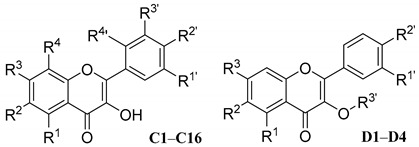												
Compd.	R^1^	R^2^	R^3^	R^4^	R^1′^	R^2′^	R^3′^	R^4′^	Alone	with FLC ^[a]^	FICI	Mode of Interaction
**C1**	OH	H	OH	H	H	H	H	H	>64	4	0.094	Synergism
**C2**	OH	H	OH	H	OH	OH	H	H	>64	4	0.094	Synergism
**C3**	OH	H	OH	H	H	OH	H	H	>64	8	0.125	Synergism
**C4**	OH	H	OH	OH	H	OH	H	H	>64	16	0.188	Synergism
**C5**	H	H	OH	H	OH	OH	OH	H	>64	2	0.078	Synergism
**C6**	OH	H	OH	H	H	OH	H	OH	>64	32	0.313	Synergism
**C7**	OH	H	OH	H	OH	OH	OH	H	>64	2	0.078	Synergism
**C8**	OH	H	OCH_3_	H	OCH_3_	OCH_3_	H	H	>64	64	0.563	Addition
**C9**	OH	H	OH	Isopentenyl	H	OCH_3_	H	H	>64	64	0.563	Addition
**C10**	OCH_3_	H	OCH_3_	H	OCH_3_	OCH_3_	H	H	>64	64	0.563	Addition
**C11**	H	OH	H	H	H	H	H	H	>64	32	0.313	Synergism
**C12**	H	H	OH	H	OH	OH	H	H	>64	32	0.313	Synergism
**C13**	H	H	H	H	OCH_3_	OCH_3_	H	H	>64	>64	2.000	Indifferent
**C14**	H	H	H	H	OH	OH	H	H	>64	4	0.094	Synergism
**C15**	OH	H	OH	H	OCH_3_	OH	H	H	>64	32	0.313	Synergism
**C16**	OH	H	Rha-O	H	OH	OH	H	H	>64	4	0.094	Synergism
**D1**	OCH_3_	H	OCH_3_	–	OCH_3_	OCH_3_	CH_3_	NA	>64	64	0.563	Addition
**D2**	OH	H	OH	–	OH	OH	Rha	NA	>64	64	0.563	Addition
**D3**	OH	H	OH	–	OH	OH	Glu	NA	>64	64	0.563	Addition
**D4**	OH	H	OH	–	OH	OH	Gal	NA	>64	>64	2.000	Indifferent
**BE**	OH	OH	OH	H	H	H	H	H	64	4	0.125	Synergism

^[a]^ MIC_80_ value of compound in combination with 8.0 μg/mL FLC. –: no substitution.

**Table 4:** The antifungal activity of some selected compounds by checkerboard microdilution assay. (MIC80, μg/mL, 48 h).

Compd.	Alone	Combination		FICI	Interaction
		FLC	Compd.		
**A4**	>64	1	0.125	0.0088	Syn
**B4**	16	1	1	0.0703	Syn
**B5**	>64	1	0.125	0.0088	Syn
**C5**	>64	0.25	4	0.0332	Syn
**C14**	>64	0.25	8	0.0645	Syn
**BE**	64	0.25	4	0.0645	Syn

FICI, fractional inhibitory concentration index for combination; Syn, synergism, FICI ≤ 0.5; Add, addition, 0.5 ≤ FICI ≤ 1; Indif, indifferent, 1 ≤ FICI ≤ 4; Anta, antagonism FICI > 4.

**Table 5:** Results of IFD and molecular dynamics simulations between **BE**, **A4**, **B4**, **B5**, **C5**, **C7** and possible targets. (Energy, kcal/mol).

Target Name	BE		A4		B4		B5		C5		C7	
	Docking Score	Energy	Docking Score	Energy	Docking Score	Energy	Docking Score	Energy	Docking Score	Energy	Docking Score	Energy
1,3-β-d-glucan synthase catalytic subunit	−7.33	−37.47	−7.46	−45.47	−6.72	−44.47	−6.35	−38.00	−6.38	−36.81	−7.35	−32.99
1,3-β-d-glucan-UDP glucosyltransferase	−8.30	−31.06	−7.52	−34.49	−8.28	−34.35	−7.51	−32.49	−8.32	−40.55	−6.33	−28.13
Chitin synthase 2	−6.81	−38.68	−5.70	−37.76	−7.27	−38.68	−7.46	−35.59	−6.98	−38.92	−7.22	−33.34
δ-sterol 5-desaturase	−4.83	−29.12	−4.14	−18.05	−4.17	−26.52	−4.84	−17.60	−4.83	−23.20	−6.43	−22.12
14-α demethylase	−11.57	−25.30	−8.38	−22.48	−9.50	−22.47	NA *	NA *	−10.26	−34.70	−8.25	−12.21
Glycosyl-phosphatidylinositol protein	−6.05	−37.28	−6.44	−39.00	−6.28	−35.09	−5.65	−35.19	−7.81	−46.74	−7.08	−46.33
Agglutinin-like protein 3	−5.87	−32.40	−5.28	−31.78	−6.02	−28.93	−6.28	−33.38	−7.11	−31.20	−7.63	−35.62

NA * indicates that the complex is unable to bind spontaneously in MDs.

## Data Availability

Data is contained within the article.
